# Genome-Wide Association Studies and Prediction of Tan Spot (*Pyrenophora tritici-repentis*) Infection in European Winter Wheat via Different Marker Platforms

**DOI:** 10.3390/genes12040490

**Published:** 2021-03-27

**Authors:** Quddoos H. Muqaddasi, Roop Kamal, Vilson Mirdita, Bernd Rodemann, Martin W. Ganal, Jochen C. Reif, Marion S. Röder

**Affiliations:** 1 Leibniz Institute of Plant Genetics and Crop Plant Research (IPK), Corrensstraße 3, D-06466 Stadt Seeland OT Gatersleben, Germany; kamal@ipk-gatersleben.de (R.K.); reif@ipk-gatersleben.de (J.C.R.); roder@ipk-gatersleben.de (M.S.R.); 2 European Wheat Breeding Center, BASF Agricultural Solutions GmbH, Am Schwabeplan 8, D-06466 Stadt Seeland OT Gatersleben, Germany; vilson.mirdita@basf.com; 3 Julius-Kühn-Institute (JKI), D-38104 Braunschweig, Germany; bernd.rodemann@julius-kuehn.de; 4 TraitGenetics GmbH, Am Schwabeplan 1b, D-06466 Stadt Seeland OT Gatersleben, Germany; martin.ganal@sgs.com

**Keywords:** wheat, tan spot, GWAS, QTL, genome-wide prediction

## Abstract

Tan spot, caused by the fungus *Pyrenophora* *tritici-repentis* (*Ptr*), is a severe foliar disease of wheat (*Triticum* *aestivum* L.). Improving genetic resistance is a durable strategy to reduce *Ptr*-related losses. Here, we dissected *Ptr*-infection’s genetic basis in 372 European wheat varieties via simple sequence repeats (SSRs) *plus* 35k and 90k single nucleotide polymorphism (SNP) marker platforms. In our phenotypic data analyses, *Ptr* infection showed a significant genotypic variance and a significant negative correlation with plant height. Genome-wide association studies revealed a highly quantitative nature of *Ptr* infection and identified two quantitative trait loci (QTL), viz., *QTs.ipk-7A* and *QTs.ipk-7B*, which imparted 21.23 and 5.84% of the genotypic variance, respectively. Besides, the *Rht-D1* gene showed a strong allelic influence on the infection scores. Due to the complex genetic nature of the *Ptr* infection, the potential of genome-wide prediction (GP) was assessed via three different genetic models on individual and combined marker platforms. The GP results indicated that the marker density and marker platforms do not considerably impact prediction accuracy (~40–42%) and that higher-order epistatic interactions may not be highly pervasive. Our results provide a further understanding of *Ptr*-infection’s genetic nature, serve as a resource for marker-assisted breeding, and highlight the potential of genome-wide selection for improved *Ptr* resistance.

## 1. Introduction

Tan spot, also known as the yellow leaf spot, is a severe disease of wheat worldwide. Caused by the fungal pathogen *Pyrenophora tritici-repentis*, (*Ptr*; Died.) anamorph *Drechslera tritici-repentis* (*Dtr*; Died.) Shoem. (syn. *Helminthosporium tritici-repentis*), the *Ptr* infection is mainly diagnosed by tan-colored necrotic lesions with yellow margins that are often surrounded by chlorotic haloes on susceptible wheat leaves. Mature lesions have a dark area in the center. With time, the lesions become larger and often fuse, resulting in the decrease of leaves’ photosynthetic surface area. Consequently, dead leaf tissue areas translate to plant stress and eventually yield loss [1]. *Ptr*-associated yield losses prove more detrimental- especially at adult stages, e.g., between growth stages BBCH-45 and -65, i.e., mid of booting to mid of flowering [2]. The yield losses—that may reach up to 50%—are mainly attributed to the reduction in (1) leaf area index, (2) dry matter accumulation, and (3) the number of reproductive tillers [3,4]. Besides, reduced kernel size, kernel weight, and the number of kernels per ear were reported to be the main drivers of *Ptr*-associated yield losses [5]. The fungal spores overwinter in the previous wheat crop’s stubble residue and reproduce in the following spring and summer [6,7]. In high disease pressure, the tan spot can also infect ears and eventually kernels, leading to the seeds’ red- or pink-smudge disease [1]. No- or minimum-tillage practices were reported to result in high disease infestation where infected stubble or kernels from previous cropping seasons act as a disease inoculum [6,7,8]. The absence of cover crops and weedicide application coupled with susceptible wheat lines, a favorable environment (i.e., rainy summer), and no-tillage help the fungus flourish. The fungus produces at least three necrotrophic effectors (NEs), viz., *Ptr*-ToxA, *Ptr*-ToxB, and *Ptr*-ToxC (for reviews, see [9,10]). The NEs—previously called host-selective toxins—are recognized by host sensitivity (*S*) genes and lead to dominant susceptibility [9]. The lack of fungal NEs recognition by the host (wheat) results in an incompatible interaction and leads to resistant wheat lines. Based on the three NEs mentioned earlier, the *Ptr* isolates have been classified into eight races [11].

Farm or agronomic management practices, e.g., primary and secondary tillage, crop rotation, and cultivar mixtures, are suitable measures to prevent disease-associated damages [7]. However, the accompanying monetary demerits may prevent their continuous use, especially by smallholdings. On the other hand, the timely use of broad-spectrum foliar fungicides—especially in times of high pressure of multiple allied diseases, e.g., *Septoria tritici* blotch and *Stagonospora nodorum* blotch—can help prevent the disease spread and benefit economically by higher yields. Nevertheless, extensive fungicide applications may result in a high pathogen evolution rate and are not sustainable. Hence, improving the resistance by exploiting genetics is deemed as a durable strategy for sustainable gains.

Three dominant *S*-genes, viz., *Tsn1*, *Tsc1*, and *Tsc2*, have been identified on wheat chromosomes 5BL, 1AS, and 2BS, respectively [12,13,14]. *Tsn1*—the first and the only gene cloned thus far for the tan spot necrosis [15]—interacts with *Ptr*-ToxA, whereas *Tsc1* and *Tsc2*—for tan spot chlorosis—were reported to interact with *Ptr*-ToxC and *Ptr*-ToxB, respectively. Besides, four tan spot resistance (*tsr*) qualitative genes, viz., *tsr*2–*tsr*5 (syn. *tsn*2–*tsn*5), were reported [16,17,18,19]. The presence of *S*-genes or absence of *tsr*-genes leads to cultivar susceptibility. The tan spot’s genetic architecture has been studied mainly via bi-parental mapping studies primarily to identify large-effect loci (reviewed in [10]), and, as a result, virtually tens of quantitative trait loci (QTL) have been identified, many of which correspond to the already identified *S*- or *tsr*-genes [20]. Genome-wide association studies (GWAS) that exploit the allelic diversity in diverse lines have also been performed to elucidate the tan spot’s genetic basis. Gurung et al. [21] were the first to show the potential of GWAS for tan spot to identify QTL in diverse spring wheat landraces. Since then, several studies report the QTL associated with both seedling and adult plant tan spot susceptibility and resistance in different panels comprising diverse landraces, breeding lines, and elite released varieties of both spring and winter wheat habitats [22,23,24,25,26,27,28]. Genome-wide prediction (GP) is a slightly different but related approach that exploits genome-wide markers’ effects rather than only the significant loci to predict the individual’s genetic merit for the trait under selection [29]. Recent GP studies on wheat diseases suggest its promising potential in breeding for improved quantitative resistance [30,31,32].

Here, we dissected the genetic basis of the *Ptr* infection in a diverse panel of recently registered 372 European wheat varieties previously studied only with the simple sequence repeats markers. We improved the molecular data by fingerprinting the varieties with high-density 35k and 90k single-nucleotide polymorphism marker arrays. We identified large-effect *Ptr*-associated QTL by combining all marker platforms suggesting the use of improved marker density. In addition, we studied the prospects of genome-wide selection (GS) by checking the efficiency of the individual marker platform to predict *Ptr*-infection’s genetic value. The GP accuracies showed that GS could be performed to improve quantitative genetic resistance and that marker platform, or marker density, does not substantially impact prediction accuracy.

## 2. Materials and Methods

### 2.1. Collection and Analyses of the Phenotypic Data

A panel (GABI) of European wheat lines comprising 372 varieties (358 winter type; 14 spring type) was evaluated for tan spot (*Ptr*) infection/resistance. The *Ptr*-infection’s phenotypic data were gathered from three replications in two environments, with each environment considered a location-by-year combination. The inoculation was performed by using a mixture of various German tan spot field isolates. Ten flag and ten first leaves were evaluated from every genotype in each replication for the percentage of *Ptr* infected area. The average percent *Ptr* infected area from all leaves was taken to represent each variety’s overall *Ptr* score in each replication. A detailed protocol for inoculation at various growth stages and disease scoring methodology is provided in Kollers et al. [23]. The field trials were conducted in α-lattice design. More details about the field trials, agronomic practices, climatic conditions, and calculation of the across-replications arithmetic entry means of each genotype in individual environments have been described previously [23]. Since disease data are generally skewed, we performed the square-root transformation on the individual environment’s data to improve the statistical normality. The normality of the phenotypic data was assessed via the Shapiro–Wilk test at P=0.001.

To compute the across-environment individual variance components of the genotype, environment, and the residuals, the following linear mixed-effect model was used by assuming all effects except the intercept as random:

(1) $$y_{ij}=\mu +g_{i}+e_{j}+\varepsilon_{ij}$$

where $y_{ij}$ is the phenotypic value (arithmetic mean) of the $i^{th}$ genotype in the $j^{th}$ environment, μ is the common intercept term, $g_{i}$ is the effect of the $i^{th}$ genotype, $e_{j}$ is the effect of the $j^{th}$ environment, and $\varepsilon_{ij}$ is the corresponding residual term as $\varepsilon \sim N\left(0,I\sigma_{\varepsilon }^{2}\right)$ with I and $\sigma_{\varepsilon }^{2}$ being the identity matrix and residual variance. The broad-sense heritability $\left(H^{2}\right)$ was calculated as:

(2) $$H^{2}=\frac{\sigma_{g}^{2}}{\sigma_{g}^{2}+\left(\frac{\sigma_{\varepsilon }^{2}}{nE}\right)}$$

where $\sigma_{g}^{2}$ and $\sigma_{\varepsilon }^{2}$ denote the genotype and residuals’ variance components, respectively, and nE represents the number of environments. The best linear unbiased estimations (BLUEs) across environments were calculated by assuming the intercept and genotype effects fixed in Equation (1). Since plant height (PH; cm) and heading date (HD; the number of days counted after 1st January) are purposed as morphological escape traits for various diseases [32,33], we retrieved data from previously published multiple-environment studies on the same panel [34,35]. We calculated the genetic correlations among all the traits based on their BLUEs computed across environments.

### 2.2. Collection and Analyses of the Genotypic Data

All 372 wheat varieties were genotyped with marker platforms, viz., microsatellites (simple sequence repeats; SSRs), and single nucleotide polymorphism (SNP) arrays. In total, the SSR genotyping resulted in 732 markers with 782 scorable genetic loci representing 3178 (2581 mapped and 597 unmapped) alleles, as described previously [23]. For SNP genotyping of the panel, two state-of-the-art marker platforms, viz., 35k Affymatrix breeders’ array and 90k Illumina iSELECT array were employed which generated 35,143 and 81,587 markers (p), respectively. Besides, we genotyped the whole panel with functional markers for the candidate genes, such as photoperiodism (*Ppd-D1*) and reduced height (*Rht-B1* and *Rht-D1*). Detailed information about the primer design for the candidate genes is given in Kollers et al. [23]. The SSR markers’ genetic positions were taken from the International Triticeae Mapping Initiative (ITMI) DH mapping population described in Sorrells et al. [36]. On the other hand, SNP markers from both 35k and 90k arrays were anchored onto the physical map of wheat (RefSeq *v*1.1), and the physical position of the markers and their corresponding information, e.g., location, gene-ID, and gene-length (start and end positions) were retrieved from Sun et al. [37]. In total, of the 35k and 90k SNP arrays, 26,236 (74.65%) and 60,638 (74.32%) makers were physically mapped onto the chromosomes. The SNP markers from both arrays *plus* the SSRs and candidate-gene markers’ scores were combined, which resulted in an n×p matrix of 372 × 119,966 and subjected to the quality check. The quality criteria were implemented to remove the markers with a minimum of 0.05 minor allele frequency and >5% missing or heterozygous calls; the remaining missing or heterozygous calls were imputed with the mean value of both alleles.

### 2.3. Genome-Wide Association Studies

Genome-wide association studies (GWAS) were performed on data taken from the individual environment and markers (both SSRs and SNPs) passing the quality criteria and the functional-gene markers. Let n be the varieties and p the predictor marker genotypes. Following Yu et al. [38], a standard linear mixed-effect model was used to perform GWAS as:

(3) y=1μ+Eτ+Xβ+Pv+Zu+ε

where y is the column vector of adjusted means of each genotype calculated in the individual environment, μ is the common intercept, τ, β,v,u, and ε are the vectors of the individual environment, markers, population structure (principal components), polygenic background, and the error effects, respectively; E,X,P, and Z are the corresponding design matrices. In the model, μ,τ, β, and v were assumed to be fixed while u and ε as random with $u\sim N\left(0,G\sigma_{u}^{2}\right)$ and $\varepsilon \sim N\left(0,I\sigma_{\varepsilon }^{2}\right)$. The n×n variance-covariance additive relationship matrix (G) was calculated from an n×p matrix $W=\left(w_{ik}\right)$ of marker genotypes (being 0, 1, or 2) as:

(4) $$G=\frac{\sum_{k=1}^{p}\left(w_{ik}-2p_{k}\right)\left(w_{jk}-2p_{k}\right)}{2\sum_{k=1}^{p}p_{k}\left(1-p_{k}\right)}$$

where $w_{ik}$ and $w_{jk}$ are the profiles of the $k^{th}$ marker for the $i^{th}$ and $j^{th}$ variety, respectively; $p_{k}$ is the estimated frequency of one allele in $k^{th}$ marker, described as a second solution in VanRaden [39]. Since population stratification and familial relatedness can severely impact the power to detect the real marker-trait associations (MTA) in GWAS, different methods were used to correct for population stratification and relatedness viz., (1) multiple linear regression (naïve), (2) correction of population structure by the first three principal components (*PC*_[1–3]_), (3) correction of familial relatedness via genomic relationship matrix G, and (4) correction of both population structure and familial relatedness by *PC*_[1–3]_ and G. It is expected that using both *PC*s and G in the model better corrects for the false positives. The models described above were compared by plotting expected versus observed $-\log_{10}\left(P\right)$ values in the form of a quantile-quantile (qq) plot. The most conservative model was determined by checking how well the observed $-\log_{10}\left(P\right)$ values aligned with the expected.

To declare the MTA, a liberal false discovery rate (FDR) to account for multiple testing was applied at α=0.20 [40]. As described in Utz et al. [41], the percentage of the adjusted genotypic variance (pG) explained by all QTL was determined as:

(5) $$pG=\left(\frac{R_{adj}^{2}}{H^{2}}\right)\times 100$$

where, $R_{adj}^{2}$ was calculated as $R_{adj}^{2}=R^{2}-\left(\frac{z^{\prime }}{N-z^{\prime }-1}\right)\left(1-R^{2}\right)$ by fitting the MTA $\left(z^{\prime }\right)$ in the order of their descending *P*-values in a multiple linear regression model; $R^{2},\;N$, and $H^{2}$ denote the regression coefficient, number of observations, and the broad-sense heritability calculated in Equation (2), respectively. The pG explained by the individual MTA was accordingly calculated from their sum of squares. The identified QTL were named based on recommended rules for gene or QTL symbolization in wheat (available online: https://wheat.pw.usda.gov/ggpages/wgc/98/Intro.htm, accessed on 18 January 2021).

### 2.4. Genome-Wide Predictions

Genome-wide prediction (GP) studies were performed by using three different models with different assumptions, viz., genomic best linear unbiased predictions (GBLUP), Bayesian alphabet B (BayesB), and reproducing kernel Hilbert space regression (RKHSR). GBLUP is a standard robust parametric procedure to predict the total genetic value of the trait under consideration by exploiting additive effects of the markers assuming equal variances [39,42]. It is a linear model of the form:

(6) y=1μ+g+ε

where y is the column vector of BLUEs calculated across environments in Equation (1), μ is a common intercept, and g=Zu; the Zu and ε are explained in Equation (3).

Since the distribution of marker variances across loci is not always equal, the BayesB model, which is of the form:

(7) y=1μ+Xβ+ε

utilizes a scaled inverse Chi-squared $\left(\chi^{-2}\right)$ distribution on the marker variances. This circumvents the problem of equal variance by assuming a prior distribution (π; the prior proportion of non-zero effects) that yields a scaled *t*-distribution for marker effects by using both shrinkage and variable selection methods. Here, y is explained in Equation (6), and X,β and ε are explained in Equation (3). Following Pérez and de los Campos [43], the prior distribution can be modeled as:

(8) $$p\left(\beta_{j},\sigma_{\beta }^{2},\pi \right)=\left\{\prod_{k}\left[\pi N\left(\beta_{jk}|0,\sigma_{\beta }^{2}\right)+\left(1-\pi \right)1\left(\beta_{jk}=0\right)\right]\chi^{-2}\left(\sigma_{\beta_{jk}}^{2}|df_{\beta },S_{\beta }\right)\right\}\;\times B(\left(\pi |p_{0},\pi_{0}\right)\times G\left(S_{\beta }|r,s\right)$$

where N and B denote normal and beta densities; β and $\sigma_{\beta }^{2}$ represent the vector of regression coefficients and respective variance. To set the hyper-parameters, we implemented the built-in procedures of the BGLR statistical package [43].

The RKHSR is a semiparametric method that accounts for the additive as well as epistatic interactions among loci [44]. It is of the same form as GBLUP (Equation (6)) with the assumption that g=Kα, and thus—by using Gaussian kernel—can be represented as:

(9) y=1μ+Kα+ε

where y,μ, and ε are the same as described in Equation (6), and α is the vector of random effects with $\alpha \sim N\left(0,\;K\sigma_{\alpha }^{2}\right)$. Here, K is n×n symmetric positive-definite matrix and is defined as $K_{ij}=e^{\left(-h\times \frac{d_{ij}^{2}}{p}\right)}$ where, $K_{ij}$ represents the measured relationship between the $i^{th}$ and $j^{th}$ variety based on their marker profiles, $d_{ij}^{2}$ is the Euclidean distance between the $i^{th}$ and $j^{th}$ variety and h is the bandwidth parameter. To determine the optimum h, three different values as h=0.5×(1/5,1,5) were tested in a five-fold cross-validation scenario, and the value representing the highest accuracy was chosen.

We evaluated the genome-wide prediction accuracy $\left(r_{GP}\right)$ of all models using a five-fold cross-validation scenario, as described in Muqaddasi et al. 2020. Briefly, the varieties were randomly divided into five subsets; four were used as the training set to estimate the remaining test set’s genetic values. The accuracy of prediction was defined as the Pearson’s product-moment correlation between the observed (y) and predicted $\left(\hat{y}\right)$ genetic values standardized by the square-root of the broad-sense heritability as $r_{GP}=\frac{cor\left(y,\;\hat{y}\right)}{H}$. Since the cross-validation runs were repeated for 100 cycles, mean and standard deviation values were calculated to show the individual prediction model’s performance. Unless stated otherwise, all calculations were performed in R software [45] mainly by using lme4 [46] and rrBLUP [47] packages.

## 3. Results

### 3.1. Phenotypic Data Analyses Reveal Significant Genetic Variation and a Strong Negative Correlation of Tan Spot Infection with Plant Height

**Figure 1:**
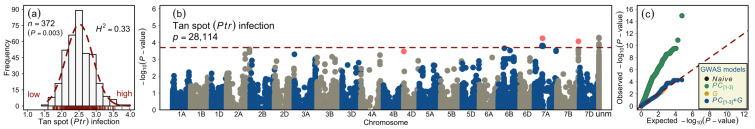
Phenotypic distribution and summary of the genome-wide association studies (GWAS) of tan spot infection in European winter wheat varieties. (**a**) Histogram of the square-root transformed tan spot *Pyrenophora tritici-repentis* (*Ptr*) infection scores on wheat varieties. (**b**) Manhattan plot showing the distribution of marker significance $\left[-\log_{10}\left(P-\mathrm{values}\right)\right]$ along the wheat chromosomes. The correction for population stratification was performed by using the first three principal components ($PC_{\left[1-3\right]}$ ) and an additive relationship matrix (*G*) in the linear mixed-effect model. The red dashed line marks the false discovery rate (FDR) threshold (α=0.20) to detect marker-trait associations. (**c**) Quantile-quantile (qq) plot showing the distribution of observed versus expected (red dashed line) $-\log_{10}\left(P-\mathrm{value}\right)$ based on the naïve model (the general linear model without correction for population structure), $PC_{\left[1-3\right]}$ model (the population structure corrected with first three *PC*s), the *G* model (population structure corrected with a genomic relationship matrix), and the ${PC}_{\left[1-3\right]}+G$ model (population structure corrected with both *PC*s and the *G* matrix). The pink highlighted markers in the Manhattan plot designate the representative markers. The color code of different models is given in the figure legend. *n* = the number of varieties; *p* = significance value of the Shapiro–Wilk normality test; $H^{2}$ = broad-sense heritability; *P* = the number of quality marker loci; unm = the unmapped markers.

The tan spot (*Ptr*) infection assessment on 372 wheat varieties registered primarily for European markets was performed in replicated field trials. The phenotypic data from the individual environment was square-root transformed (Table S1). We observed a moderate (r=0.20) but significant Pearson’s product-moment correlation between both environments’ adjusted means (Figure S1a). The ANOVA showed that both genotype and environment variance was significantly (P<0.001) larger than zero (Table 1). The best linear unbiased estimations (BLUEs) calculated across environments approximated a statistically normal distribution (Shapiro–Wilk P=0.003) and ranged from 1.58 to 3.97 with a mean of 2.51 and median of 2.48; the 1st and the 3rd quantiles amounted to 2.23 and 2.77, respectively (Figure 1a and Figure S1b). The broad-sense heritability amounted to 0.33, suggesting a sizeable environmental variance; this is expected due to uneven disease pressures in different environments.

We retrieved data for plant height and heading date from previously published studies to observe their influence on the tan spot infection. We observed a highly significant negative Pearson’s product-moment correlation of tan spot infection with PH while a moderate negative correlation with HD (Figure S1c). This indicates that taller and later heading plants—on average—escape *Ptr* infection and that shorter plants are more susceptible to the disease infestation.

### 3.2. GWAS Reveals Medium- to Large-Effect Loci Controlling the Tan Spot

We performed GWAS based on environment-specific phenotypic scores and the genotypic matrix comprising the full set of quality markers (p=28,114) that were combined from SSRs, two SNP arrays, and candidate-gene markers. It was shown earlier that, on this panel, increasing the marker density results in improved detection of the marker-trait associations (MTA) [32]. In this study, the GWAS model correcting both population structure and genomic relationships (Figure S2) could sufficiently control spurious MTA detection (Figure 1b,c). Our GWAS resulted in the detection of two quantitative trait loci (QTL) and, in total, identified 28 MTA, of which 19 were distributed on chromosome 7A (*QTs.ipk-7A*) and 1 on chromosome 7B (*QTs.ipk-7B*). The remaining eight MTA were unmapped and, therefore, no chromosomal and physical position was assigned to them (Table 2 and Table S2). Since *QTs.ipk-7A* harbored several MTA, only one marker with the highest $-\log_{10}\left(P\right)$ value and genotypic variance $\left(pG_{adj}\right)$—hereafter termed as a representative marker—was taken to represent the QTL. The representative markers of the QTL, viz., *QTs.ipk-7A,* and *QTs.ipk-7B* imparted $pG_{adj}$ = 21.23 and 5.84%, respectively. The total $pG_{adj}$ imparted by all MTA amounted to 25.79%.

**Figure 2:**
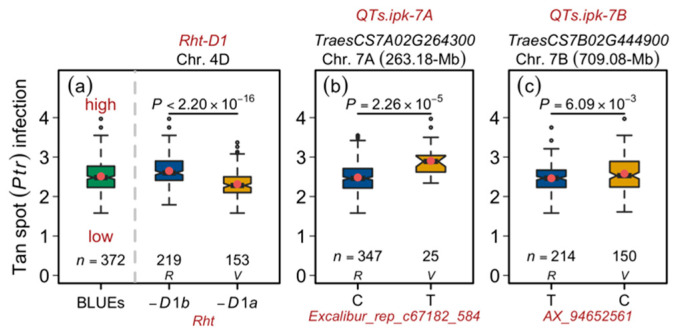
Allelic influence of the tan spot-associated quantitative trait loci (QTL) in European wheat. (**a**) Distribution of the best linear unbiased estimations (BLUEs; left panel) to compare the allele-wise distribution of the *Rht-D1* alleles. (**b**) Allelic distribution of the representative marker for tan spot-associated locus *QTs.ipk-7A*. (**c**) Allelic distribution of the representative marker for tan spot-associated locus *QTs.ipk-7B*. The first, second, and third rows in the figure header show the QTL name, the gene-ID corresponding to the most significant marker in the QTL, and the QTL’s chromosome and physical position of the representative marker. The *x*-axis shows the representative marker names and their alleles. *n* = number of varieties harbored by the corresponding panel; *P* = significance value of Welch two-sample *t*-test; *R =* reference (major) allele; and *V* = variant (minor) allele.

As observed in the phenotypic data analyses, a highly significant negative correlation of *Ptr* infection was observed with the plant height, suggesting that taller plants escape the disease infestation. Nevertheless, our GWAS—albeit setting a liberal MTA detection threshold (FDR) of 0.20—did not identify the *Rht* genes. The FDR value for *Rht-D1* was, however, 0.25 and, therefore, being close to the threshold and frequent (*Rht-D1a* = 0.41; *Rht-D1b* = 0.59) in the European wheat germplasm, we investigated its genetic/allelic influence on the tan spot. The findings concurred with the phenotypic analyses where the impact of *Rht-D1a* (wild-type; tall allele) was significantly greater than *Rht-D1b* (dwarfing allele; short allele) in terms of reducing the *Ptr* infection (Figure 1b and Figure 2a). Similarly, allele-wise phenotypic distribution showed a significant difference between the varieties harboring the reference (major) and variant (minor) allele of the representative marker for the 7A-QTL *QTs.ipk-7A* (gene-ID = *TraesCS7A02G264300*; Table 2, Figure 2b). The same was true for another small- or medium-effect locus *QTs.ipk-7B* on chromosome 7B (gene-ID = *TraesCS7B02G444900*; Table 2; Figure 2c). Since the physical interval of the *QTs.ipk-7A* is large (~36-Mb), it is difficult to identify a single causative gene. Nevertheless, the large effect of 7A-QTL explaining >20% genotypic variance merits its future use for gene cloning and downstream molecular and functional analyses.

**Figure 3:**
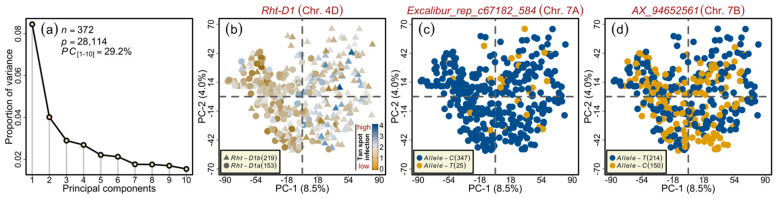
Principal component (*PC*) analysis on the wheat marker loci combined from the SSR alleles, functional candidate-gene markers, 35k, and 90k single nucleotide polymorphism arrays. (**a**) Scree plot showing first ten *PC*s and their corresponding proportion of variance. (**b**) Two-dimensional scatterplot showing the absence of pronounced clustering among the varieties, except those based on the *Rht-D1* alleles. (**c**) Scatterplot showing the lack of clustering among the varieties based on the *QTs.ipk-7A* representative marker alleles. (**d**) Scatterplot showing the absence of clustering among the varieties based on the *QTs.ipk-7B* representative marker alleles. Color codes are given in the respective sub-figure’s legend/s. *n* and *p* denote the number of varieties and the marker genotypes used in the analysis, respectively.

The extent of linkage disequilibrium (LD), the non-random association between different loci, plays a vital role in GWAS. The panel under investigation has been previously examined for the LD via different marker platforms [48,49]. In addition, the population structure and related parameters have been published earlier [49]. Here, to observe the alleles’ distribution in the investigated germplasm, we performed *PC* analyses based on singular value decomposition, as described previously [49]. The first ten *PC*s accounted for 29.2% of the total variation (Figure 3a). A two-dimensional scatterplot of the first two *PC*s for *Rht-D1* alleles showed a clear distribution of both alleles on the opposite sides of the central axis (Figure 3b). However, the large-effect QTL on chromosome 7A (i.e., *QTs.ipk-7A*)—the minor allele of which was present in only 6.7% of the varieties—showed no clear pattern (Table 2; Figure 3c).

### 3.3. Genome-Wide Prediction Studies Show That Marker Density, Marker Platform, and Genetic Models Do Not Substantially Influence the Prediction Accuracies

**Figure 4:**
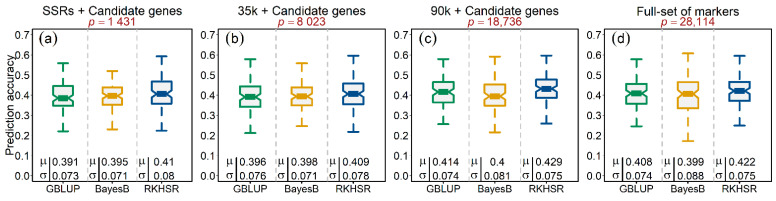
Accuracy of genome-wide prediction (GP) of tan spot (*Ptr*) infection in wheat. The figure header represents four different GP scenarios, viz., (**a**) GP based on SSR alleles and candidate genes, (**b**) GP based on quality 35k SNPs and candidate genes, (**c**) GP based on quality 90k SNPs and candidate genes, and (**d**) GP based on markers combined from every platform. The GP accuracy assessment is based on three models, viz., genomic best linear unbiased prediction (GBLUP), Bayesian alphabet B (BayesB), and reproducing kernel Hilbert space regression (RKHSR). The GP accuracies were evaluated through 100 random five-fold cross-validation cycles. Symbols μ and σ denote the mean accuracy and standard deviation of the corresponding model, respectively.

To observe the influence of individual marker platforms on the genome-wide prediction accuracies of *Ptr* infection, we tested three different models making different assumptions in this study, thus creating four scenarios as (1) SSR alleles, (2) 35k SNP array, (3) 90k SNP array, and (4) the full set of markers altogether. In every scenario, we incorporated the functional candidate-gene markers as well. The mean prediction accuracies resulting from the five-fold cross-validation scenario of *Ptr* infection generally produced similar results (~40%) across all three tested model scenarios, i.e., the GBLUP model that accounted for the main additive effects of markers assuming equal variances, BayesB by assuming unequal marker variances, and RKHSR that accounted for both additive and the epistatic interactions among the loci (Figure 4a–d). Overall, the 90k platform outperformed every other scenario with higher (1–2%) prediction accuracies. The RKHSR resulted in relatively better prediction accuracy than the GBLUP and BayesB, suggesting—albeit not highly prevalent—the presence of epistatic interactions for the *Ptr* infection.

## 4. Discussion

### 4.1. A Parallel Exploitation of Genetic Variation and Morphological Escape Traits Can Help Improve the Tan Spot Resistance in Wheat

A significant genetic variation for the traits under selection provides a substantial impetus in improving breeding programs’ genetic gains. However, especially for disease traits, besides the genotypic variation, the presence of a large and significant genotype-by-environment interaction is virtually a norm mainly because (1) the disease pressures are uneven across environments, and (2) the environmental effects are very unpredictable. We evaluated 372 registered wheat varieties in replicated field trials and observed significant genotypic variation for tan spot (*Ptr*) infection. However, due to large and significant genotype-by-environment interaction, we observed a moderate broad-sense heritability that amounted to 0.33. Recently, based on multiple environment trials, Juliana et al. [30] reported similar moderate broad-sense heritability estimates for the tan spot adult plant resistance in wheat.

Coupled with significant genetic variation, certain easy-to-score morphological traits have been purposed to escape disease infestations not only for the tan spot but also for other diseases, e.g., *Fusarium* head blight and *Septoria tritici* blotch [32,33,48]. We observed a highly significant negative correlation of plant height and a moderate negative but significant correlation of heading date with tan spot infection. Based on previous studies and this study, it seems that the major genes for plant height (*Rht*) or photoperiodism (*Ppd*) may show a pleiotropic effect on the disease traits.

### 4.2. The Influence of Rht-D1 and QTs.ipk-7A on the Tan Spot for Marker-Assisted Selection

We identified two significant QTL associated with tan spot on chromosomes 7A and 7B at the 263.18 and 709.08-Mb positions. Although previous studies on both bi-parental and diverse populations have reported tan spot-associated loci on chromosomes 7A and 7B, none of them resulted in identifying QTL imparting >20% of the genotypic variance [22,23,24,25,26,27,28]. Recently, Liu et al., [20] in a meta-QTL study, identified one QTL on chromosomes 7A (116.1–133.2-Mb) and two on 7B (21.0–34.0-Mb and 614.2–622.8-Mb). The physical distances of the QTL identified in our study from the meta-QTL are large and, given an extensive linkage disequilibrium in wheat, they may be considered novel. Also, the comparison of markers and their corresponding positions is not possible, mainly due to different marker systems and maps (physical and/or genetic). Marker-assisted selection (MAS) is profitable per unit time and cost only when the trait-tagged markers impart considerable genotypic variation. Therefore, due to sizeable genotypic variance, i.e., 21.23%, the 7A-QTL is of interest for MAS. The 7B-QTL explained 5.84% of the genotypic variance and can be considered a second target for MAS.

Besides, as shown in Figure 1b and Figure 2a, the functional marker for the candidate gene *Rht-D1*—although it did not pass the significance threshold—showed a relatively large effect on the tan spot infection score. This indicates that the MAS based on the *Rht-D1a*, i.e., the wild-type or tall allele for plant height, may help select for tan spot resistance. Since the tan spot infection is more lethal at later plant growth stages (e.g., BBCH-45–65) [2,4], the relatively taller plant selection should help escape the disease infestation. Consistent with this observation, the genotypes harboring *Rht-D1a* were more resistant than those bearing *Rht-D1b* (Figure 2a). Semi-dwarf or short-statured plants are, on the other hand, desired in breeding programs to achieve higher stem/stand strength. This warrants the use of genes other than *Rht-D1a* to tailor plant height. The frequency of *Rht-B1b*—as reported previously—is shallow in European varieties [34], which was perhaps why it was not identified as significantly associated with the tan spot in our study. Nonetheless, the selection of *Rht-B1b* to reduce height may not seem advisable given the similar effects of both genes on several other traits. For this purpose, other *Rht*-genes (e.g., *Rht8* or *Rht24*) may be used to fine-tune the plant height for improved lodging resistance in breeding programs [50,51].

### 4.3. Genome-Wide Prediction Accuracy Reveals the Prospects of Genome-Wide Selection for Tan Spot Resistance

Improving qualitative disease resistance by selecting for or against major genes or QTL is a resource and time-efficient measure. However, most disease genes are responsive only against one or a few pathogen races and lack a broad-spectrum application [52]. Moreover, the practical difficulty and costs become co-extensive while pyramiding several QTL in an elite background and, therefore, inadvertently affect the breeding operations. Also, relying on only one or a few large-effect genes can result in the acceleration of pathogen evolution. For long-term sustainable genetic gains, improving quantitative resistance is deemed a durable strategy. Therefore, instead of concentrating on only large-effect loci, using the total genetic value predicted by both small- and large-effect loci helps select lines with relatively broad-spectrum resistance.

In our study, we observed a highly quantitative genetic nature of the tan spot where the significantly associated markers—albeit considerably improving marker density—in total, explained only 25.79% of the genotypic variance. Markers that do not cross significance thresholds in GWAS, e.g., *Rht-D1*, are usually not used for MAS. Therefore, instead of concentrating only on large-effect loci, genome-wide prediction (GP) of the total genetic value of tan spot based on small- and large-effect markers is a holistic tactic to improve the broad-spectrum resistance. We evaluated the GP accuracy for tan spot resistance by modeling the loci’s additive effects assuming equal variances, unequal variances, and epistatic interaction. In line with a previous study, the mean GP accuracies calculated across 100 cycles and five-fold cross-validation scenarios amounted to ~40%, with virtually no statistically significant difference between the models [30]. Although epistatic interactions were previously reported to be pervasive in self-pollinating species like wheat [53], we observed only a slight increase in the prediction accuracy by modeling epistatic interactions.

Based on the hypothesis that marker platform and thus marker density influences the outcome of GP accuracy, we evaluated all three models on all marker platforms, viz., SSR, 35k, and 90k SNP arrays individually and marker loci combined from all platforms. However, increasing the marker density did not result in any significant increase in GP accuracy. This finding is in line with previous reports where GP accuracy was not influenced above a certain number of markers [31,32], underlining that all marker platforms are almost equally efficient to predict tan spot infection. In practical breeding, nevertheless, the usefulness of GP may be hampered by shifts in the virulence spectrum of the pathogen in different environments or breeding target zones.

## Figures and Tables

**Table 1:** ANOVA for tan spot in European wheat varieties.

	Df	Sum Sq	Mean Sq	F-Value	Pr(>F)	Sig.	$\sigma^{2}$	SD
Genotype	371	113.03	0.3	1.49	6.48 × 10^−5^	***	0.050	0.224
Env.	1	260.42	260.4	1273.89	<2.00 × 10^−16^	***	0.700	0.836
Residuals	371	75.84	0.2				0.204	0.452

Df = degree of freedom; Sq = squares; Sig. = significance code; $\sigma^{2}$ = variance; SD = standard deviation; Env. = environment; *** = significant at the 0.001 probability (*P*) level.

**Table 2:** The quantitative trait loci (QTL) and markers associated with the wheat tan spot infection from the combined set of candidate genes, simple sequence repeats (SSRs), 35k, and 90k marker platforms.

QTL	Marker	Chr.	Pos. (bp)	|log_10_(*P*)|	MAF	$pG_{adj}$
*QTs.ipk-7A*	*Ex_c37521_670*	7A	246258333	3.78	0.06	18.57
*QTs.ipk-7A*	*wsnp_Ra_c12708_20281439*	7A	246258437	3.78	0.06	18.57
*QTs.ipk-7A*	*BS00067759_51*	7A	247662756	3.78	0.06	18.57
*QTs.ipk-7A*	*AX_94420810*	7A	247662758	3.82	0.06	18.75
*QTs.ipk-7A*	*Ex_c6348_1205*	7A	249279881	3.78	0.06	18.57
*QTs.ipk-7A*	*Excalibur_c15904_1331*	7A	250037145	3.78	0.06	18.57
*QTs.ipk-7A*	*wsnp_Ex_c4819_8600618*	7A	252176134	3.78	0.06	18.57
*QTs.ipk-7A*	*RAC875_c109483_523*	7A	254386961	3.78	0.06	18.57
*QTs.ipk-7A*	*wsnp_Ex_c26560_35803210*	7A	254386961	3.78	0.06	18.57
*QTs.ipk-7A*	*wsnp_be471272A_Ta_2_1*	7A	260905626	3.78	0.06	18.57
***QTs.ipk-7A***	***Excalibur_rep_c67182_584***	**7A**	**263177287**	**4.25**	**0.07**	**21.23**
*QTs.ipk-7A*	*Tdurum_contig10174_155*	7A	263177287	4.25	0.07	21.23
*QTs.ipk-7A*	*RAC875_rep_c81362_344*	7A	272339544	3.78	0.06	18.57
*QTs.ipk-7A*	*AX_94706782*	7A	272997798	3.82	0.06	18.75
*QTs.ipk-7A*	*wsnp_Ex_rep_c102317_87512660*	7A	272997798	3.78	0.06	18.57
*QTs.ipk-7A*	*BS00036422_51*	7A	275545643	3.78	0.06	18.57
*QTs.ipk-7A*	*wsnp_Ra_c13009_20690735*	7A	275919716	3.78	0.06	18.57
*QTs.ipk-7A*	*wsnp_Ex_c5330_9422106*	7A	275920469	3.78	0.06	18.57
*QTs.ipk-7A*	*BS00066695_51*	7A	282368577	3.78	0.06	18.57
***QTs.ipk-7B***	***AX_94652561***	**7B**	**709082422**	**4.07**	**0.40**	**5.84**

Chr. = chromosome name; Pos. (bp) = physical position of the corresponding marker in base-pairs; |log_10_(*P*)| = negative log transformed significance (*P*) value of the corresponding marker; MAF = minor allele frequency; $pG_{adj}$ = percentage of adjusted genotypic variance imparted by the corresponding marker. The bold markers are the representative QTL descrbied in the text.

## Data Availability

Data is contained within the article and supplementary files.

## Supplementary Materials

The following are available online at https://www.mdpi.com/2073-4425/12/4/490/s1, Figure S1: Distribution and correlation of tan spot (*Ptr*) resistance with plant height and heading date, Figure S2: Genomic relationship matrix, Table S1: List of genotypes, BLUEs, and associated markers, Table S2: List of tan spot resistance associated QTL.
