# Supplementary material for: Genome-Wide Association Studies and Prediction of Tan Spot (Pyrenophora tritici-repentis) Infection in European Winter Wheat via Different Marker Platforms
Source: Genes (Basel). 2021 Mar 27;12(4):490. doi: 10.3390/genes12040490 (PMC8103242; doi:10.3390/genes12040490)
Supplement: Supplementary file 1 [file genes-12-00490-s001.zip › genes-1133958-Supplementary figures.pdf]

genes

MDPI

Supplementary figures

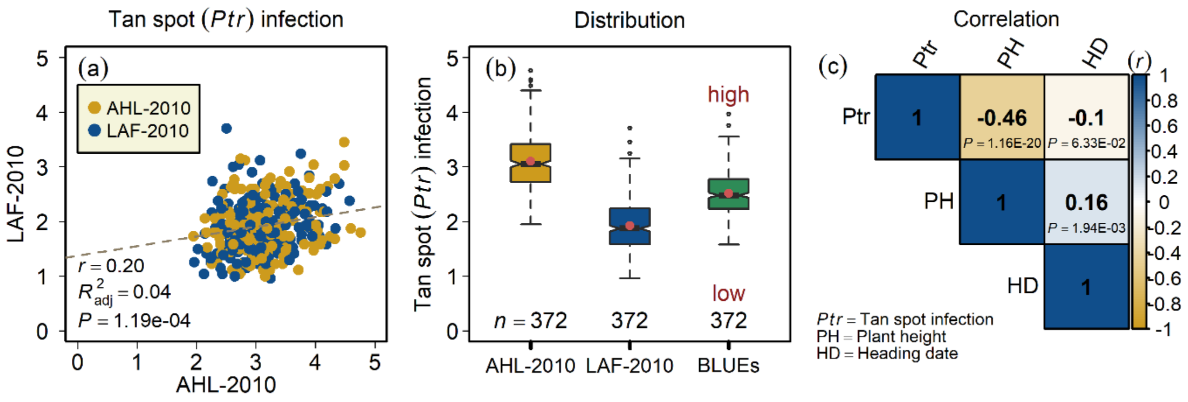

Figure S1. Distribution of tan spot (*Ptr*) resistance in wheat. (**a**) Association between the square root transformed adjusted means of *Ptr* resistance calculated in the environments AHL-2010 and LAF-2010. (**b**) Distribution of the adjusted means and best linear unbiased estimations (BLUEs) of tan spot infection calculated across environments. (**c**) Pearson product-moment correlation among tan spot infection, plant height, and heading date. r = Pearson's product moment correlation; R^2_{adj} = ajusted squared regression coefficient; P = significance of the respective correlation; n = number of varieties, respectively.

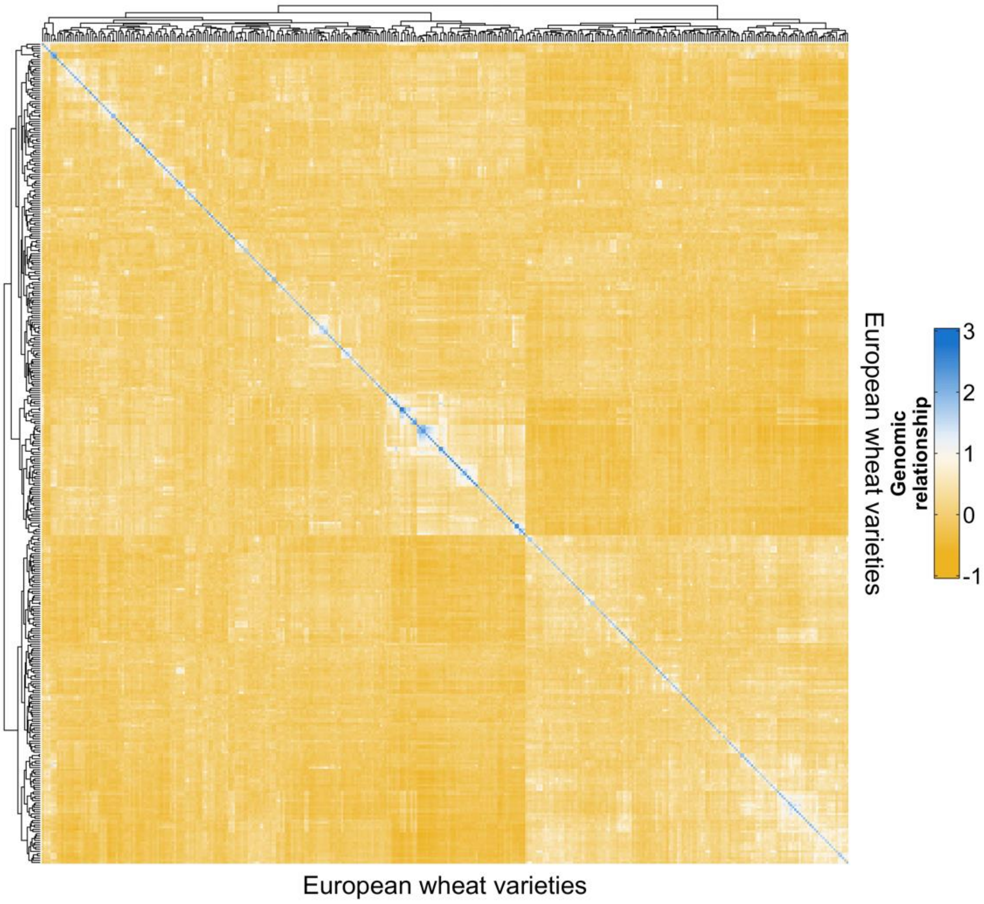

Figure S2. Genomic relationship matrix (VanRaden 2008–2nd solution) based on 28,114 marker genotypes on 372 European registered wheat varieites. Color code is given in the figure legend.
